# Association Between Age and Race and Cervical Cancer Stage

**DOI:** 10.1097/og9.0000000000000138

**Published:** 2025-12-04

**Authors:** Nicole Santos, Jamie Kim, Katie Hsu, Edward Gemson, Eloise Chapman-Davis, Denise Howard, Lauren Mount, Rulla M. Tamimi

**Affiliations:** Weill Cornell Medicine, New York, New York; Stony Brook University, New York, New York; and Department of Obstetrics and Gynecology and Department of Population Health Science, Weill Cornell Medicine, New York, New York.

## Abstract

**OBJECTIVE:**

The American College of Obstetricians and Gynecologists recommends cervical cancer screening for people with a cervix between the ages of 21 and 65 with cytology and human papillomavirus cotesting starting at age 30. These broad guidelines are intended to improve cervical cancer prevention and early detection but apply only to those with normal results. Surveillance guidelines for those with abnormal results are different and may not be widely followed. There is concern that older women and women of color may not benefit from these guidelines to the same extent as younger White women. To address a gap in the literature by conducting a retrospective cross-sectional study using the National Cancer Database to assess the relationship between age and race and late-stage cervical cancer diagnosis.

**METHODS:**

We conducted a cross-sectional study using data obtained from the National Cancer Database for the years 2004–2022. Women between 21 and 85 years of age with a known cervical cancer stage were included in this study. Age, race, and the combined effect of age and race were the exposures of interest. The outcome of interest was the diagnosis of late-stage (III and IV) compared with early-stage (I and II) cervical cancer. The association among race, ethnicity, and late-stage cervical cancer diagnosis was examined with multivariate-adjusted logistic regression models adjusted for insurance status, facility type, region, education, and income. A likelihood ratio test was used to test for the interaction between age and race.

**RESULTS:**

From 2004 to 2022, there were 102,131 early-stage (62.6%) and 61,076 late-stage (37.4%) cervical cancers in the National Cancer Database. Women 65 years of age and older had significantly higher odds of being diagnosed with late-stage cervical cancer (multivariate odds ratio [OR_MV_] 1.6, 95% CI, 1.5–1.7) compared with younger women; this was similar for all racial and ethnic groups. Non-Hispanic Black women had 16% (OR 1.2, 95% CI, 1.1–1.2) higher odds of a late-stage diagnosis of cervical cancer compared with non-Hispanic White women. Conversely, Hispanic (OR_MV_ 0.8, 95% CI, 0.8–0.9) and non-Hispanic Asian/Pacific Islander (OR_MV_ 0.9, 95% CI, 0.9–1.0) women had lower odds of a late-stage diagnosis compared with non-Hispanic White women. Non-Hispanic Black women 65 year of age and older had the highest odds of a late-stage diagnosis of cervical cancer compared with non-Hispanic White women 64 years or age or younger (OR_MV_ 1.9, 95% CI, 1.7–2).

**CONCLUSION:**

These data support the hypothesis that women 65 years of age and older are at increased risk for late-stage cervical cancer compared with younger women. These findings suggest that the increased risk at later ages may be attributable to lack of adherence to screening guidelines or recommendations for surveillance and management of screening abnormalities. These findings have important implications for upstream structural drivers of these disparities and the need for equity-driven strategies that address the systemic barriers affecting older women across all racial and ethnic groups, especially non-Hispanic Black women.

## Abstract 2

Women 65 years of age or older are more at risk for late-stage cervical cancer, and current screening and surveillance guidelines may need to be re-evaluated.

Cervical cancer is a significant public health concern in the United States, with an estimated 13,960 new cases of late-stage diagnosis and 4,310 deaths in 2023.^1^ Although the median age at diagnosis is 50, women over the age of 65 account for approximately 20% of all cervical cancer cases in the United States, and incidence rates from the National Cancer Institute Surveillance, Epidemiology, and End Results program show sustained high incidence rates after the age of 65 for non-Hispanic Black and Hispanic women.^2^ Current screening guidelines^3,4^ recommend discontinuing routine cervical cancer screening at the age of 65 for women who have already completed adequate prior screening, defined as three consecutive negative cytology results or two consecutive negative cotesting results within 10 years before stopping screening, because new cases of cancer past the age of 65 rarely occur in women who have regularly been getting testing and screening.

Adequate cervical cancer screening has significantly reduced cervical cancer mortality.^5^ However, screening utilization varies across racial and ethnic groups, with lower rates observed among non-Hispanic Black women and women from other racial and ethnic backgrounds compared with non-Hispanic White women.^6^ Studies also indicate that screening in non-Hispanic Black women is equivalent to that in other races, but guideline-recommended follow-up after detecting abnormalities in cervical cancer screening is lower among non-Hispanic Black women.^7^ Furthermore, recent data^2^ indicate that age–incidence curves for cervical cancer vary by race and ethnicity: Incidence rates for White women peak at age 39 and decline with age, whereas for non-Hispanic Black women, incidence rates peak at age 79. For Hispanic and Asian women, incidence rates tend to plateau and remain high after age 40. Because race is a social construct, we hypothesize that historic processes and institutional processes are associated with race and ethnicity. Marginalized racial groups may experience discrimination that can affect their exposure to factors related to cervical cancer risk and access to preventive health care along the cancer care continuum.

Women diagnosed with cervical cancer after 65 likely do not meet recommended guidelines for discontinuation of screening or surveillance.^8,9^ Overall, the prevalence of having an abnormal screening based on cytology alone is about 4–7%,^10,11^ whereas abnormal screening based on cotesting (cytology or human papillomavirus [HPV]) is higher, about 17%.^10^ According to the 2019 ASCCP guidelines, women who have been treated for high-grade cervical lesions should undergo posttreatment surveillance, not routine screening, with HPV cotesting at 3-year intervals for at least 25 years, with surveillance strategies tailored to their individual risk based on histologic findings and HPV status. Given that cervical cancer incidence rates for non-White women in the United States remain high after age 65 and that the current cervical screening guidelines recommend discontinuation of screening at this age for women at low risk, we hypothesize that older age would be associated with late-stage cervical cancer among non-White women and especially among non-Hispanic Black women, for whom we see the greatest disparities. This study aims to assess whether age, race, or both are associated with late-stage diagnosis of cervical cancer.

## METHODS

To assess cervical cancer outcomes, data were obtained from the National Cancer Database for the years 2004–2022. A joint program sponsored by the American Cancer Society and the American College of Surgeons Commission on Cancer, the National Cancer Database is a hospital-based cancer registry that gathers data from more than 1,500 U.S. hospitals, collecting more than 70% of all newly diagnosed malignancies in the United States.^12^ Incident cases of cancer were assessed; therefore, patients had no prior cervical cancer diagnosis, and the date of diagnosis referenced is their first or only diagnosis. The National Cancer Database population consists of patients who received any part of cancer care (treatment or diagnosis) at a Commission on Cancer–accredited cancer program, and the data registry includes standardized data on tumor characteristics, including American Joint Committee on Cancer clinical and pathologic staging, number of lymph nodes evaluated, surgical margin status, tumor size, differentiation (grade), and sociodemographic factors such as age, sex, and insurance status at both the census and facility levels. Professional registrars from the National Cancer Database enter individual-level deidentified data using a standard approach for oncology registry entry to ensure data quality across sites. A Participant User File for cervical cancer was obtained from the National Cancer Database and contained the data used in this study. Women 21 to 85 years of age and older who were diagnosed with cervical cancer were identified in the Participant User File and included in this study.

Age was dichotomized (64 years and younger, 65 years and older) to assess the age at which the current cervical cancer screening guidelines recommend women to stop screening. Race and ethnicity were categorized as non-Hispanic White, non-Hispanic Black, non-Hispanic Asian/Pacific Islander, non-Hispanic American Indian/Alaska Native, non-Hispanic Other, and Hispanic. Individuals with no reported information on race or ethnicity were excluded.

Women were considered to have cervical cancer if the anatomic site of origin of the cancer was the endocervix, exocervix, overlapping lesion of cervix uteri, and cervix uteri.^13^ The National Cancer Database Analytic stage group reported the patient's stage at the initial diagnosis. National Cancer Database Analytic stage group was determined by the reported pathologic stage group, and if not reported, clinical stage group was used. The stage of cervical cancer diagnosis was then dichotomized into early stage (stages I and II) and late stage (stages III and IV).

We considered the following relevant covariates for this study: education, household income, residence area, insurance status, and facility type. The variables education and household income were determined by comparing the patient's ZIP code at the time of diagnosis to the 2020 American Community Survey data for the years 2016–2020. Education was classified as percentage of the population without completion of high school and was reported as four ordinal categorical variables: less than 5.0%, less than 5.0–9.0%, 19.1–15.2%, and 15.3% or more. The classification is the number of adults in the patient's ZIP code who did not complete high school and is divided into equal-proportioned quartiles across all U.S. ZIP codes. This may represent the subject's educational attainment. Household income was adjusted for inflation in 2020 and considered as four ordinal categorical variables: less than $46,277, $46,277–$57,856, $57,857–$74,062, and $74,063 or more. Residence area was categorized as metro, urban, and rural. Insurance status indicates the main insurance provider at time of initial diagnosis and was reported as five nominal categorical variables: not insured, private insurance/managed care, Medicaid, Medicare, and other government. Facility type was categorized by structural characteristics of the reporting facility and considered as four nominal categorical variables: Community Cancer Program, Comprehensive Community Cancer Program, academic/research program, and Integrated Network Cancer Program. It should be noted that facility type for women 0–39 years of age were suppressed.

Descriptive statistics were used to examine frequency distributions of the categorical variables by stage of cervical cancer diagnosis. Logistic regression was used to assess the relationships between the three exposures of interest—age, race and ethnicity, and the combined effect of age and race/ethnicity—with the outcome stage at diagnosis for cervical cancer. Multivariate logistic regression models were used to estimate the crude and adjusted odds ratios (ORs) and their 95% CIs. A primary aim of this study was to evaluate the interaction between age and race and ethnicity. A likelihood ratio test was used to assess the statistical significance of the combined effect of age and race and ethnicity and the interaction of non-Hispanic Black women compared with all other races. A value of *P*=.05 was interpreted to be statistically significant. R 4.4.3 was used for all statistical analyses. This study was determined to be exempt from IRB approval under the Biomedical Research Alliance of New York IRB No. 22-12-425-380 because it is secondary research for which consent is not required.

## RESULTS

This study included 163,207 women diagnosed with cervical cancer; 62.6% (102,131 of 163,207) of women with cervical cancer were diagnosed with early-stage disease, and 37.4% (61,076 of 163,207) were diagnosed with late-stage cancer. Approximately 34.7% of women aged 64 years of age and younger and 48.9% of women 65 years or age and older were diagnosed with late-stage cervical cancer (Table 1). Cancer stage distribution varied by race and ethnicity, with non-Hispanic Black women having a higher percentage of late-stage cancer (43.8%) compared with non-Hispanic White women (36.7%) and Hispanic women (34.8%) having the lowest percentage of late-stage cancer (Table 1).

**Table 1.** Demographic Characteristics of Cervical Cancer Cases by Stage Group in the National Cancer Database (2004–2022) (N=163,207)

	Early Stage* (n=102,131)	Late Stage† (n=61,076)
Age (y)		
64 and younger	86,169 (84)	45,828 (75)
65 and older	15,962 (16)	15,248 (25)
Race and ethnicity		
Non-Hispanic American Indian/Alaska Native	561 (0.5)	344 (0.6)
Non-Hispanic Asian/Pacific Islander	4,761 (4.7)	2,588 (4.2)
Non-Hispanic Black	14,053 (14)	10,932 (18)
Hispanic	15,188 (15)	8,115 (13)
Non-Hispanic, none of the above	770 (0.8)	367 (0.6)
Non-Hispanic White	66,798 (65)	38,730 (63)
Residence area		
Metro	82,331 (81)	49,166 (80)
Urban	11,685 (11)	7,293 (12)
Rural	4,232 (4.1)	2,646 (4.3)
Not reported	3,883 (3.8)	1,971 (3.2)
Education (% of population completing high school)		
15.3% and higher	22,998 (23)	15,298 (25)
9.1–15.2%	25,346 (25)	16,094 (26)
5.0–9.0%	26,524 (26)	14,894 (24)
Less than 5.0%	16,396 (16)	8,177 (13)
Not reported	10,867 (11)	6,613 (11)
Household income		
Less than $46,277	20,093 (20)	14,149 (23)
$46,277–$57,856	22,573 (22)	13,913 (23)
$57,857–$74,062	23,993 (23)	13,877 (23)
$74,063 and more	24,563 (24)	12,508 (20)
Not reported	10,909 (11)	6,629 (11)
Insurance		
Medicaid	20,904 (20)	15,655 (26)
Medicare	16,748 (16)	14,979 (25)
Not insured	7,364 (7.2)	5,718 (9.4)
Other government	1,325 (1.3)	646 (1.1)
Private insurance	53,578 (52)	22,698 (37)
Not reported	2,212 (2.2)	1,380 (2.3)

Values are n (%).

* Early-stage cervical cancer defined as stages I and II.

† Late-stage cervical cancer defined as stages III and IV.

In the crude model, women 65 years of age and older had 1.8 (OR 1.8, 95% CI, 1.8–1.8) times the odds of late-stage cervical cancer diagnosis compared with women 64 years of age and younger. However, after adjustment for multiple potential confounders, the OR was attenuated to 1.6 (multivariate OR [OR_MV_] 1.6, 95% CI, 1.5–1.7; Table 2).

**Table 2.** Multivariable-Adjusted Odds Ratios for Late-Stage* Cervical Cancer

Characteristics	Adjusted† OR	95% CI
Age (y)		
64 and younger	1.0 (REF)	
65 and older	1.6	1.5–1.7
Race and ethnicity		
Non-Hispanic White	1.0 (REF)	
Non-Hispanic American Indian/Alaska Native	1.0	0.9–1.2
Non-Hispanic Asian/Pacific Islander	0.9	0.9–1.0
Non-Hispanic Black	1.2	1.1–1.2
Hispanic	0.8	0.8–0.8
Non-Hispanic, none of the above	0.8	0.7–0.9
Residence area		
Metro	1.0 (REF)	
Urban	1.0	1.0–1.0
Rural	1.0	1.0–1.1
Not reported	0.9	0.9–1.0
Education (% of population completing high school)		
15.3% and higher	1.0 (REF)	
9.1–15.2%	1.0	1.0–1.0
5.0–9.0%	0.9	0.9–1.0
Less than 5.0%	0.9	0.9–0.9
Not reported	1.5	0.9–2.7
Household income		
Less than $46,277	1.0 (REF)	
$46,277–$57,856	1.0	0.9–1.0
$57,857–$74,062	0.9	0.9–1.0
$74,063 and more	0.9	0.9–0.9
Not reported	0.6	0.3–1.1
Insurance		
Medicaid	1.0 (REF)	
Medicare	0.9	0.8–0.9
Not insured	1.1	1.0–1.1
Other government	0.7	0.6–0.7
Private insurance	0.6	0.6–0.6
Not reported	0.8	0.8–0.9

OR, odds ratio; REF, referent.

* Late-stage cervical cancer defined as stages III and IV.

† Multivariate adjusted for other variables in the table.

In crude models, non-Hispanic Black women had 1.3 (OR 1.3, 95% CI, 1.3–1.4) times the odds of late-stage cervical cancer diagnosis compared with non-Hispanic White women. However, in multivariate models, non-Hispanic Black women had 1.2 (OR 1.2, 95% CI, 1.1–1.2; Table 2) times the odds of late-stage cervical cancer diagnosis compared with non-Hispanic White women. In multivariate models, non-Hispanic Asian Pacific Islander (OR_MV_ 0.9, 95% CI, 0.9–1.0; Table 3) and Hispanic (OR_MV_ 0.8, 95% CI, 0.8–0.8; Table 2) women had significantly lower odds of late-stage cervical cancer compared with non-Hispanic White women.

**Table 3.** Adjusted* Odds Ratios and 95% CIs for the Association Between Age and Race and Late-Stage† Cervical Cancer in the National Cancer Database for the Years 2004–2022

	Non-Hispanic White (n=105,528)	Non-Hispanic Black (n=24,985)	Non-Hispanic Asian/Pacific Islander (n=7,349)	Hispanic (n=23,303)	Non-Hispanic American Indian/Alaska Native (n=905)	Non-Hispanic, None of the Above (n=1,137)	All Racial and Ethnic Groups
64 y of age and younger	1.0‡ (REF)	1.2 (1.1–1.2)	0.9 (0.9–1.0)	0.8 (0.8–0.9)	1.0 (0.9–1.2)	0.8 (0.7–0.9)	**(1.0)**§ **REF**
65 y of age and older	1.6 (1.5–1.7)	1.9 (1.7–2.0)	1.5 (1.3–1.6)	1.3 (1.3–1.5)	1.2 (0.8–1.7)	1.4 (1.1–1.9)	**1.6 (1.5–1.7)**
All ages	*1.0‖ (REF)*	*1.2 (1.1–1.2)*	*0.9 (0.9–1.0)*	*0.8 (0.8–0.8)*	*1.0 (0.9–1.2)*	*0.8 (0.7–0.9)*	

REF, referent.

* Adjusted for the following variables (as categorized in Table 1): insurance status, facility type, residence area, education, and household income.

† Late-stage cervical cancer is stages III and IV. Early-stage cervical cancer is stages I and II.

‡ Reference group for cross-classified analyses of race and ethnicity and age is non-Hispanic White women 64 years of age and younger. *P* value for the multiplicative interaction effects of age and race (*P*=.25).

§ Reference group for analyses of age category (*bold*) is all women 64 years of age and younger.

‖ Reference group for analyses of race and ethnicity (*italic*s) is non-Hispanic White women.

Given the attenuation between age and race and ethnicity and risk of late-stage cervical cancer after adjustment for covariates, we aimed to better understand how socioeconomic factors were associated with late-stage cervical cancer. Compared with women with Medicaid, privately insured women had a 42% lower (OR 0.6, 95% CI, 0.6–0.6) odds of being diagnosed with late-stage cervical cancer. Higher income and education were also both associated reduced odds of late-stage cervical cancer. These results highlight the influence of social determinants of health on late-stage cervical cancer diagnosis.

Table 3 illustrates the adjusted OR and 95% CI for the association between the joint effect of age and race and late-stage diagnosis of cervical cancer. Non-Hispanic Black women aged 64 years of age and younger had 1.2 (OR_MV_ 1.2, 95% CI, 1.1–1.2; Table 3) times the odds of late-stage cervical cancer diagnosis compared with non-Hispanic White women 64 years of age and younger. Non-Hispanic Asian/Pacific Islander and Hispanic women 64 years of age and younger had decreased odds of late-stage diagnosis of cervical cancer compared with non-Hispanic White women 64 years of age and younger (non-Hispanic Asian/Pacific Islander: OR_MV_ 0.9, 95% CI, 0.9–1.0; Table 3) (Hispanic: OR_MV_ 0.8, 95% CI, 0.8–0.9; Table 3).

Older age was associated with increased odds of late-stage cervical cancer across all racial and ethnic groups (Table 3). Although non-Hispanic Black women 65 years of age and older had the highest odds of late-stage cervical cancer compared with younger non-Hispanic White women, the interaction was not statistically significant (Table 3; *P* for interaction=.25).

## DISCUSSION

Our results demonstrate that age and race are important demographic characteristics that are associated with late-stage diagnosis of cervical cancer. Cervical cancer typically develops decades after exposure to HPV, with the precancerous phase lasting approximately 15–20 years. This provides a long time window for women to receive appropriate screening and management of precancerous lesions. However, given our findings, disparities in surveillance must exist for older and non-Hispanic Black women.

We found that across all racial groups, women 65 years of age and older were at an increased odds of late-stage cervical cancer compared with younger women. Our finding, which looks at more recent data from a nationwide registry, are consistent with that of older studies,^14–16^ highlighting that late-stage diagnoses persist despite recent efforts aimed at increasing awareness of cervical cancer screening. Our results suggest that either older women are not being adequately screened in later ages or older women with previously abnormal Pap tests or positive high-odds HPV tests are not being adequately managed and surveilled. Current screening guidelines suggest that if a woman 65 years of age and older has been adequately screened before age 65 and did not have abnormal screening, she should discontinue cervical cancer screening. The rationale for this is that if a woman has not had any indication of abnormal cytology or precancerous lesions rooted in HPV infection, she is not at high odds for future cervical cancer, and the benefits of continued screening are minimal. However, the guidelines for discontinuation of cervical cancer screening at age 65 are based on the assumption of adequate prior screening without any abnormal screening. Recent data indicate that up to 25% of women between the ages of 45 and 64 are not being adequately screened.^17^ In addition, these guidelines do not pertain to women who have had an abnormal screening or history of precancerous lesions. For women previously diagnosed with CIN 2+, additional routine screening should continue for at least 25 years after the appropriate management of the precancerous lesion, even if this extends beyond age 65 years.

Our results also demonstrated that non-Hispanic Black women are more likely to be diagnosed with late-stage cervical cancer compared with women of all other races, consistent with that previous studies.^16,18,19^ Compared with non-Hispanic White women, non-Hispanic Black women experience a 41% higher incidence of cervical cancer and are twice as likely to die of this disease.^20^ It has been suggested that the later stage at diagnosis and mortality disparity observed may be attributable to different histologies of cervical cancer. Black women are more likely than White women to be diagnosed with adenocarcinoma, which has a worse prognosis than other types of cervical cancer.^21–23^ In addition, there are racial and ethnic differences in the incidence rates of HPV, the primary causal factor of cervical cancer. The National Health and Nutrition Examination Survey, which looked at the prevalence of HPV among adults 18–69 years of age in the United States from 2011 to 2014, found that the prevalence of high-risk genital HPV was highest among non-Hispanic Black adults for both men and women.^24^ Furthermore, a recent study^6^ noted that even when controlling for insurance status and site of care, non-Hispanic Black patients had the lowest rates of screening use (53.2%), whereas screening use was higher among Hispanic (65.4%) and Asian/Pacific Islander (66.5%) women compared with non-Hispanic White women (63.5%).

Taken together, our results suggest that older women, in particular older Black women, may not be receiving adequate screening surveillance. Notably, women without insurance have significantly higher odds of being diagnosed with late-stage cervical cancer, suggesting that lack of access to regular screening and timely follow-up may be a critical driver of disease progression. In this context, surveillance refers to ongoing follow-up, monitoring, and management after having a positive screening. There are very limited data on rates of high-grade cervical lesions and HPV status among older individuals in recent years. A better understanding of rates of precursor lesions by age and race in recent years will help to inform strategies to support appropriate surveillance for these women who continue to be at high risk of cervical cancer even after age 65.

When we study cancer health disparities, it is important to understand the broader historical and social context when interpreting the study results. The Warnecke framework for heath disparities outlines how multiple levels of influence, from social circumstances to biologic mechanisms, can lead to differences across populations.^25^ In the present study, we were able to assess individual-level factors and neighborhood characteristics. An important finding was that having private insurance was associated with a 40% reduced risk of late-stage cervical cancer, indicating the importance for insurance as it relates to care along the cancer continuum. However, we were unable to assess more upstream factors such discrimination, other health care system differences, or other societal factors that should be considered the fundamental causes or “causes of the causes.”

This study has a few weaknesses that should be noted. First, the National Cancer Database does not capture all of the cervical cancer cases in the United States, and the majority of cases in National Cancer Database are non-Hispanic White women. The smaller sample sizes for some racial and ethnic groups reflect broader patterns of underrepresentation in cancer registries and may have limited our ability to fully characterize disparities among these populations. The National Cancer Database gathers data only on individuals diagnosed or treated at facilities recognized by the Commission on Cancer, which are more likely to be in urban areas.^18^ These are of concern because our study population may not be generalizable to the general population. Lastly, there may be other important confounders that we were unable to account for in our analyses. In addition, we did not have information on other variables of interest, including HPV status, screening history, or previous precancerous lesions. The social determinants of health variables (eg, education and income) are area-based measures that are assigned according to where the individual lives and are not based on individual-level information. These variables may not accurate capture individual-level social determinants of health. Future studies should examine these factors in contemporary cohorts to better identify points along the cancer continuum in which interventions should be focused to improve cervical cancer prevention for all women.

Given the racial, ethnic, and age disparities that arise in late-stage cervical cancer diagnosis, this study highlights the need for additional research to better understand adherence by patients and clinicians to guidelines and specifically to manage precancerous abnormalities and appropriate surveillance for women with previous precancerous cervical lesions and those with high-odds HPV. It also suggests that more resources and efforts are needed to improve the disparities related to cervical cancer prevention, which includes management of screening abnormalities such as treatment and appropriate long-term surveillance, which should extend 25 years after resolution of disease.

It is critical to identify and remove barriers to cervical cancer screening and surveillance of older women, especially those in minority groups for whom disparities in cancer risks and outcomes exist. This might include expanding educational resources for patients and clinicians in understanding new screening and management guidelines and for whom they are intended, as well as understanding the importance of long-term surveillance for women with a history of precancerous lesions. In addition, ensuring equitable and robust uptake of HPV vaccination will also be important for the prevention of cervical cancer in the future.
